# TripLexicon: prediction and analysis of gene regulatory RNA–DNA interactions

**DOI:** 10.1093/bioinformatics/btaf641

**Published:** 2025-12-01

**Authors:** Timothy Warwick, Christina Kalk, Ralf P Brandes, Marcel H Schulz

**Affiliations:** Institute for Cardiovascular Physiology, Goethe University Frankfurt, 60590 Frankfurt am Main, Hessen, Germany; German Centre for Cardiovascular Research (DZHK), Partner Site Rhein-Main, 60590 Frankfurt am Main, Germany; German Centre for Cardiovascular Research (DZHK), Partner Site Rhein-Main, 60590 Frankfurt am Main, Germany; Institute for Computational Genomic Medicine, Goethe University Frankfurt, 60590 Frankfurt am Main, Hessen, Germany; Institute for Cardiovascular Physiology, Goethe University Frankfurt, 60590 Frankfurt am Main, Hessen, Germany; German Centre for Cardiovascular Research (DZHK), Partner Site Rhein-Main, 60590 Frankfurt am Main, Germany; German Centre for Cardiovascular Research (DZHK), Partner Site Rhein-Main, 60590 Frankfurt am Main, Germany; Institute for Computational Genomic Medicine, Goethe University Frankfurt, 60590 Frankfurt am Main, Hessen, Germany

## Abstract

**Motivation:**

Non-coding RNA (ncRNA) plays a crucial role in gene regulation, including by forming sequence-specific RNA–DNA interactions at gene regulatory elements. One form of interaction takes place via the formation of RNA:DNA:DNA triple helices (triplexes). Accurate computational prediction of triplex formation from nucleotide sequences is an important tool in ncRNA research but remains somewhat inaccessible and complex. To address this, we created *TripLexicon*, a web-based interface for accessing and analyzing predicted gene regulatory RNA–DNA interactions in human and mouse.

**Results:**

Predicted interactions can be accessed from RNA-, DNA-, and region-centric perspectives. For each RNA transcript, visualizations at genome and nucleotide resolution are available, providing insight into target genes and regions, as well as putative functional domains of the transcript. Predicted target genes can immediately be subjected to ontology and pathway enrichment analysis, providing rapid insight into potential functions mediated by the RNA–DNA interactions of the queried transcript. DNA and region queries are designed to identify potentially important ncRNA interactors at sites of interest.

**Availability and implementation:**

*TripLexicon* is accessible at https://triplexicon.uni-frankfurt.de. This website is free and open to all users and there is no login requirement. All data and code is uploaded to Zenodo: https://zenodo.org/records/17143608 and the code for the webserver is available on Github: https://github.com/SchulzLab/TripLexicon.

## 1 Introduction

An ever-increasing body of evidence has demonstrated that RNA molecules are not merely messengers but can also act as gene regulatory factors. One mechanism through which RNA-based gene regulation occurs is the formation of physical interactions between RNA and DNA. One modality by which RNA and DNA may interact is via the formation of RNA:DNA:DNA triple helices, known as triplexes. Here, the single-stranded RNA binds in the major groove of the double-stranded DNA through Hoogsteen base pairing, while the DNA conformation remains intact. Hoogsteen base pairing, and therefore triplex formation, are sequence-specific (Kunkler *et al.* 2019). This has led to the hypothesis that RNA can contribute to the precise control of gene expression via sequence-specific RNA–DNA interactions. We and others have demonstrated that ncRNAs can bind to gene regulatory regions via the formation of triplexes and elicit functional consequences on associated target gene expression (Mondal *et al.* 2015, Trembinski *et al.* 2020, Leisegang *et al.* 2022, Zhang *et al.* 2022).

Because molecular methods for the identification and characterization of triplexes remain highly complex, computational prediction of triplex formation has become a key method in non-coding RNA research. Several tools exist for this purpose, with varying approaches and outputs (Warwick *et al.* 2023). Each of these require a level of computational expertise, such as familiarity with the command-line interface or R, limiting community accessibility to these methods.

To remedy this, we conceived and constructed *TripLexicon*, a web server which permits users to access and analyze predicted gene regulatory RNA–DNA interactions. Predicted interactions were computed with our previously published and validated tool *TriplexAligner* (Warwick *et al.* 2022). Interactions were predicted in human and mouse, using all annotated lncRNAs and a mixed repertoire of gene regulatory elements (see online supplementary material). Visualizations depicting the predicted interactions of ncRNAs at genome and nucleotide resolutions are available per transcript, and predicted target genes can immediately be tested for pathway and gene ontology enrichment.

*TripLexicon* is accompanied by comprehensive documentation alongside use cases and examples. Results tables and plots describing the predicted interactions of the user-supplied transcript, gene or region can be downloaded freely in several formats.

## 2 Methods

### 2.1 Triplex prediction

For details on preparation of the RNA and DNA sequences, see online supplementary material. The predicted RNA–DNA interactions hosted by the web server were predicted with a combination of *TriplexAligner* (Warwick *et al.* 2022) and *LAST* (Kiełbasa *et al.* 2011). Initially, a *LAST* database of the aforementioned DNA element sequences was created per species. These databases were subsequently used in *LAST* for the computation of local alignments with the species-appropriate lncRNA sequences, using custom triplex scoring matrices computed in the development of *TriplexAligner*. Karlin-Altschul *E*-values reflecting the resulting local alignment scores were calculated using *TriplexAligner*, with species-matched query and database sizes. This resulted in a collection of predicted RNA–DNA interactions per species, summarized by *E-value*. An SQLite database was constructed from these interactions and associated RNA and DNA metadata.

### 2.2 Input

*TripLexicon* can be queried for predicted triplexes of human and mouse. The species needs to be selected by the user at the beginning of each query. There are three different query modes to retrieve predicted triplexes from the *TripLexicon* webserver.

#### 2.2.1 RNA query

To query *TripLexicon* by RNAs, two different input options are available. The user can supply an RNA gene ID [Ensembl release 112 for human or mm39 for mouse (Harrison *et al.* 2024)], RNA gene name, or RNA transcript ID with which a summary or detailed query can be performed. The summary query will report the triplexes summarized for each transcript of the respective RNA gene, while the detailed query will report each triplex as a separate row with more detailed information.

#### 2.2.2 DNA query

The DNA query enables the user to query *TripLexicon* by DNA target elements for predicted triplex formation. Gene IDs or gene names of the target genes need to be supplied and detailed results of each triplex predicted for the respective gene will be reported including the gene name and transcript ID of the RNA partner.

#### 2.2.3 Region query

The user can supply genomic regions and predicted triplexes for DNA target elements inside the supplied regions will be returned. The region input can be provided via input fields if the query of a single region is of interest. The size of the input region is restricted to 1 million base pairs. If several genomic regions are to be investigated at the same time, a bedfile of the respective regions can be uploaded. The results will show all predicted triplexes for DNA target elements which overlap at least 50% with one of the specified region(s). The overlap between the regions is calculated with the pybedtools (v0.10.0) (Dale *et al.* 2011) functionality.

### 2.3 Output

The results of *TripLexicon* queries are predicted triplexes and appropriate metadata. The results tables are dynamic, and can be freely exported to CSV, Excel or BED files. Results tables can be sorted and searched on the web server. The output table available via the web server is restricted to the 10 000 triplexes with the lowest *E*-value for the rare cases where more triplexes are predicted. Full results tables for all predicted interactions are available for download via Zenodo: https://zenodo.org/records/17143608.

All query options result in output tables with identical columns. The RNA gene name and transcript ID are reported (“RNA” and “Transcript ID”), as well as the start and end positions of the region in the transcript forming the triplex (“Transcript Triplex Start” and “Transcript Triplex End”). The predicted DNA targets are provided as gene names (“Target gene”) and genomic coordinates of the predicted triplex site (“Target Triplex Chromosome,” “Target Triplex Start”, and “Target Triplex End”). The transcript length and DNA target element length are reported. The score, bit score and *E* value are metrics calculated by *TriplexAligner* (Warwick *et al.* 2022) to assess the statistical significance of the triplex prediction using Karlin-Altschul statistics (Karlin and Altschul 1993). The *TriplexAligner* sequence alignment and respective scoring scheme underlying the predicted interaction can be viewed by selecting “View Alignment.”

Selecting a gene name in the “RNA” column will redirect to the summary view of the respective RNA gene. This is equivalent to the results of an RNA summary query, and lists all transcripts of the respective RNA gene. Some columns differ from the ones described for detailed queries, as more information on the RNA gene and transcript is given (coordinates, biotype), and total triplexes per transcript is displayed under “Transcript Triplexcount.” Below the summary table are two plots of the transcript with the highest predicted triplex count. The first plot depicts the transcript colored according to the summed $-log_{10}(E)$ values of all its triplex predictions per nucleotide (Supplementary Documentation, available as supplementary data at *Bioinformatics* online). The second is a circos-style plot, depicting which region of the transcript interacts with which genomic loci (Supplementary documentation, available as supplementary data at *Bioinformatics* online). The corresponding view for a single transcript can be obtained by clicking on a transcript ID in any of the results tables. Additionally, predicted binding sites of the RNA in question can be viewed in the UCSC Genome Browser by selecting “Binding Sites in UCSC,” which will redirect the user to the genome browser with the binding sites loaded as a custom track.

For the detailed RNA gene and transcript query, the results page contains a button “GO enrichment of DNA gene set” which allows gene ontology (GO) enrichment of the target DNA gene set, implemented via *g: Profiler* (Kolberg *et al.* 2023). The DNA genes are restricted to the protein coding biotype. The GO enrichment results page contains one scatter plot for each of the different pathway databases [at the time of writing: GO: MF Molecular Function, GO: BP Biological Process, Reactome (REAC), KEGG and WikiPathways (WP)], displaying the term and associated statistical significance (*g: Profiler* adjusted *P* value).

More detailed information on the query in- and outputs can be found in the *TripLexicon* documentation https://triplexicon-webserver.readthedocs.io/en/latest/examples.html.

## 3 Results

### 3.1 Case study: conservation of triplex-forming RNA function

The lncRNA *KCNQ1OT1* has been reported to suppress the expression of transposable elements across the human genome via the formation of triplexes and recruitment of the heterochromatin protein HP1α (Zhang *et al.* 2022). However, the authors only presented limited findings in mouse, leading to the question of whether the mechanism is conserved between human and mouse.

**Figure 1.**
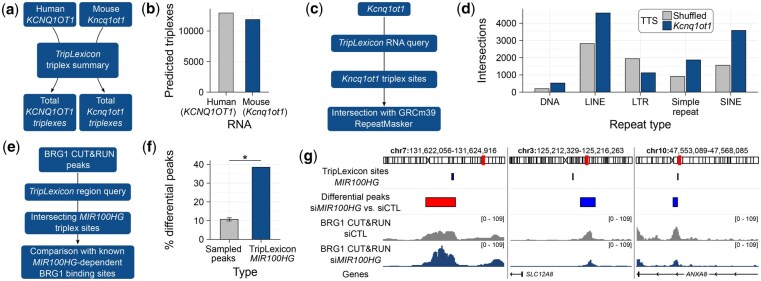
Examples of TripLexicon analyses. (a, b) Using the triplex summary function to retrieve total triplexes per RNA. (c, d) *Kcnq1ot1* triplex targets site (TTS) intersections with RepeatMasker repeats, compared with the intersection of shuffled (n=100) sites. (e) Region query workflow for retrieving triplex target sites overlapping with BRG1 CUT&RUN peaks (Oo *et al.* 2025). (f) Comparison between *MIR100HG* overlap with either differential BRG1 peaks or randomly samples (n=100) peaks. (g) *MIR100HG* triplex target sites alongside BRG1 binding sites altered upon *MIR100HG* knockdown.

To answer this using *TripLexicon*, the triplex summaries of mouse (*Kcnq1ot1*) and human (*KCNQ1OT1*) transcripts were retrieved using the RNA Query modality (Fig. 1a, also see online supplementary material). Both transcripts are predicted to form a comparable number of triplexes across the mouse and human genome (11 873 and 12 943, respectively) (Fig. 1b), indicating the triplex forming potential of the transcript is conserved between the species. To investigate whether the triplex formation of the murine transcript might have a similar function to its human counterpart, the predicted triplex target sites of *Kcnq1ot1* were retrieved from *TripLexicon*, again using the RNA query function (Fig. 1c). The mouse sites were downloaded as a BED file and intersected against RepeatMasker annotations of the GRCm39 genome. The predicted *Kcnq1ot1* binding sites showed considerable overlap with long interspersed nuclear elements (LINEs), short interspersed nuclear elements (SINEs) and simple repeats as annotated by RepeatMasker (Fig. 1d), when compared with randomly shuffled (n=100) target sites.

This application demonstrates the speed at which questions which would previously have required computational expertise and an extended length of time can be answered using *TripLexicon*. Using the web server, it could be demonstrated that the published functional importance of *KCNQ1OT1* triplexes in human cells may be conserved to mouse.

### 3.2 Case study: identifying triplex binding regions for known regulators

In a second use case, *TripLexicon* was used to identify potential triplex-mediated transcription factor binding. The protein-of-interest was BRG1, a component of the SWI/SNF complex whose localization on chromatin was recently reported to be at least partially dependent on lncRNAs (Oo *et al.* 2025).

BRG1 CUT&RUN peaks (GSE262061) were used as input to a *TripLexicon* region query (Fig. 1e, see online supplementary material), returning triplexes predicted to form in those regions. Following download as a BED file, predicted triplexes were subset to those predicted to be formed by *MIR100HG*, a lncRNA shown to impact BRG1 localization. These sites were then compared to BRG1 binding sites shown to be differentially bound following *MIR100HG* depletion (Oo *et al.* 2025). Predicted *MIR100HG* triplex sites were significantly more likely to be differential upon *MIR100HG* depletion compared to size-matched samples of all BRG1 peaks (Fig. 1f). These sites were a mixture of up- and downregulated BRG1 binding sites (Fig. 1g), indicating a highly site-specific mechanism of recruitment.

This use case demonstrates how TripLexicon can be used in conjunction with diverse epigenomic data (CUT&RUN, ChIP-seq, ATAC-seq) to explore the putative role of RNA–DNA interactions in transcription factor recruitment. This could be utilized to identify potential molecular mechanisms of lncRNA functions, or to explore candidate transcription factors whose localization may be influenced by the RNA-binding status of DNA.

## 4 Discussion

*TripLexicon* represents the only currently available resource to access and analyze pre-computed RNA–DNA interactions. By permitting users to rapidly access predicted lncRNA–DNA interactions and perform downstream analysis of target gene sets, *TripLexicon* fills a previously empty niche in the RNA research community, complementing more manual methods such as *Triplex Domain Finder* (Kuo *et al.* 2019), *LongTarget* (Wen *et al.* 2022), *3plex* (Cicconetti *et al.* 2023), and *TriplexAligner* (Warwick *et al.* 2022).

We believe that *TripLexicon* represents a valuable resource for the community for exploring the roles of lncRNAs in gene regulatory networks. *TripLexicon* allows analyses, such as those described here, to be conducted in a fraction of the time of previously published, more manual methods. The accessible nature of the web server also opens this area of research to users who may lack the requisite expertise or resources to conduct complex computational analyses.

## Supplementary Material

btaf641_Supplementary_Data

## Data Availability

All data underlying the resource have been uploaded to Zenodo under https://doi.org/10.5281/zenodo.17143607 and the code used to assemble the resource is available at https://github.com/SchulzLab/TripLexicon.
