# Supplementary material for: TripLexicon: prediction and analysis of gene regulatory RNA–DNA interactions
Source: Bioinformatics. 2025 Dec 1;41(12):btaf641. doi: 10.1093/bioinformatics/btaf641 (PMC12711254; doi:10.1093/bioinformatics/btaf641)
Supplement: btaf641_Supplementary_Data [file btaf641_supplementary_data.pdf]

Supplementary material for "TripLexicon: Prediction and analysis of gene regulatory RNA-DNA interactions"

Supplementary methods

Data processing

For both human and mouse RNA sequences, all annotated lncRNAs were taken from the respective GENCODE [Frankish et al., 2022] annotation (51,306 human lncRNAs from GENCODE v45, 21,884 murine lncRNAs from GENCODE vM35). Human DNA elements consisted of promoter regions, annotated as TSS +500/-2500 base pairs, totaling 252,893 regions, along with 2,404,861 gene-linked *EpiRegio* regulatory elements [Baumgarten et al., 2020]. Mouse promoters were designated in an identical manner, resulting in 149,101 regions. Because *EpiRegio* does not currently host mouse regulatory elements, these were instead defined using ATAC-sequencing data hosted by ENCODE [The ENCODE Project Consortium, 2012, Luo et al., 2020]. Briefly, all available raw mouse ATAC-sequencing data were downloaded and aligned to the GRCm39 genome assembly using *Bowtie2* (v2.4.5) [Langmead and Salzberg, 2012]. Peaks per sample were subsequently called from the resulting alignments using *MACS* (v3.0.0a7) [Zhang et al., 2008] with the parameters *–broad* and *–format BAMPE*. A union of the resulting peak regions was used as input to *STARE* [Hecker et al., 2023], which was used to assign peaks to genes in a distance-based manner. For both species, the appropriate genomic sequences (GRCh38/GRCm39) underlying the gene regulatory element regions were extracted using *bedtools getfasta* (v2.27.1) [Quinlan and Hall, 2010].

Triplex statistics

Tables 1 and 2 contain summary statistics of the number of triplexes for the human and mouse database, respectively. The mean, maximum and standard deviation for the number of triplexes per RNA transcripts, RNA gene and DNA gene are indicated.

Nr Triplexes per Category	Mean	Max	Std
RNA transcript	115.93	33 110	335.29
RNA gene	310.04	33 151	678.83
DNA Ensembl ID	94.48	1761	108.32

Table 1. Summary statistics for predicted triplexes in human.

Nr Triplexes per Category	Mean	Max	Std
RNA transcript	129.61	12 001	255.33
RNA gene	227.36	12 011	366.34
DNA Ensembl ID	35.37	441	31.92

Table 2. Summary statistics for predicted triplexes in mouse.

Application scenario 1

To analyze whether the triplex function of the human lncRNA *KCNQ1OT1* may be conserved to the murine equivalent *Kcnq1ot1*, *TripLexicon* was used to retrieve information on the predicted triplex formation of each transcript via the RNA Query modality. Following download of the murine target sites, the resulting CSV file was imported into *R* (v4.4.1) [R Core Team, 2024], where the regions were converted to a *GRanges* object using *GenomicRanges* (v1.56.1) *MakeGRangesFromDataFrame()* [Lawrence et al., 2013]. To conduct the intersection with transposable elements, the GRCm39 *RepeatMasker* table was downloaded from the UCSC Table Browser [Karolchik et al., 2004], read into *R* and also transformed into a *GRanges* object. Intersections were computed between the triplex sites and RepeatMasker regions using *plyranges* (v1.24.0) *FindOverlaps()* [Lee et al., 2019], with the total overlaps per repeat class calculated from the result. To compare this to background repeat presence, the triplex sites were shuffled throughout the GRCm39 genome 100 times using *regioneR* (v1.36.0) *randomizeRegions()* [Gel et al., 2016]. Each set of shuffled regions was also intersected with the *RepeatMasker* repeat regions, and the total intersections summarized per shuffle. Resulting counts were plotted using *ggplot2* (v3.5.1) [Wickham, 2016].

Application scenario 2

To examine whether triplex formation could represent a potential mechanism by which the lncRNA *MIR100HG* impacts the genome-wide localization of the transcription factor SMARCA4, published CUT&RUN peaks [Oo et al., 2025] were used as input to a *TripLexicon* Region Query. Due to the high number of peaks, and resulting total genomic coverage, this was done with three chunks of the peak BED file, and results were then

© The Author . Published by Oxford University Press. All rights reserved. For permissions, please e-mail: journals.permissions@oup.com

concatenated. The resulting *TripLexicon* results were downloaded as a CSV and imported into *R* (v4.4.1) [R Core Team, 2024]. Here, the results were subset to only those involving the lncRNA *MIR100HG*. Separately, SMARCA4 peaks from an experiment where *MIR100HG* was depleted using siRNA were read into *R*, and the *MIR100HG* triplex sites were compared against these differential peaks using *plyranges* (v1.24.0) *FindOverlaps()* [Lee et al., 2019]. Size-matched samples of peaks from the differential experiment were also taken for statistical comparison to the *MIR100HG* triplex sites. To assess the statistical significance of the association between predicted *MIR100HG* sites and differential SMARCA4 peaks after *MIR100HG* knockdown, a Fisher's Exact Test was performed in *R*, where the overall numbers of differential and non-differential peaks were compared to the number of differential and non-differential *MIR100HG TripLexicon* sites. Plots were constructed using *ggplot2* (v3.5.1).

References

N. Baumgarten, D. Hecker, S. Karunanithi, F. Schmidt, M. List, and M. H. Schulz. EpiRegio: analysis and retrieval of regulatory elements linked to genes. *Nucleic Acids Research*, 48(W1): W193–W199, May 2020. ISSN 1362-4962. doi: 10.1093/nar/gkaa382.

A. Frankish, S. Carbonell-Sala, M. Diekhans, I. Jungreis, J. E. Loveland, J. M. Mudge, C. Sisu, J. C. Wright, C. Arnan, I. Barnes, et al. GENCODE: reference annotation for the human and mouse genomes in 2023. *Nucleic Acids Research*, 51(D1): D942–D949, Nov. 2022. ISSN 1362-4962. doi: 10.1093/nar/gkac1071.

B. Gel, A. Diez-Villanueva, E. Serra, M. Buschbeck, M. A. Peinado, and R. Malinverni. regioneR: an R/Bioconductor package for the association analysis of genomic regions based on permutation tests. *Bioinformatics*, 32(2):289–291, 2016. doi: 10.1093/bioinformatics/btv562.

D. Hecker, F. Behjati Ardakani, A. Karollus, J. Gagneur, and M. H. Schulz. The adapted Activity-By-Contact model for enhancer–gene assignment and its application to single-cell data. *Bioinformatics*, 39(2), Jan. 2023. ISSN 1367-4811. doi: 10.1093/bioinformatics/btad062.

D. Karolchik, A. S. Hinrichs, T. S. Furey, K. M. Roskin, C. W. Sugnet, D. Haussler, and W. J. Kent. The UCSC Table Browser data retrieval tool. *Nucleic Acids Research*, 32(suppl_1):D493–D496, 2004.

B. Langmead and S. L. Salzberg. Fast gapped-read alignment with Bowtie 2. *Nature Methods*, 9(4):357–359, Mar. 2012. ISSN 1548-7105. doi: 10.1038/nmeth.1923.

M. Lawrence, W. Huber, H. Pagès, P. Aboyoun, M. Carlson, R. Gentleman, M. Morgan, and V. Carey. Software for Computing and Annotating Genomic Ranges. *PLoS Computational Biology*, 9, 2013. doi: 10.1371/journal.pcbi.1003118.

Lee, Stuart, Cook, Dianne, Lawrence, and Michael. plyranges: a grammar of genomic data transformation. *Genome Biology*, 20 (1):4, 2019.

Y. Luo, B. C. Hitz, I. Gabdank, J. A. Hilton, M. S. Kagda, B. Lam, Z. Myers, P. Sud, J. Jou, K. Lin, et al. New developments on the Encyclopedia of DNA Elements (ENCODE) data portal. *Nucleic Acids Research*, 48(D1):D882–D889, 2020.

J. A. Oo, T. Warwick, K. Pálfi, F. Lam, F. McNicoll, C. Prieto-Garcia, S. Günther, C. Cao, Y. Zhou, A. A. Gavrilov, et al. Long non-coding RNAs direct the SWI/SNF complex to cell type-specific enhancers. *Nature Communications*, 16(1), Jan. 2025. ISSN 2041-1723. doi: 10.1038/s41467-024-55539-6.

A. R. Quinlan and I. M. Hall. BEDTools: a flexible suite of utilities for comparing genomic features. *Bioinformatics*, 26(6):841–842, Jan. 2010. ISSN 1367-4803. doi: 10.1093/bioinformatics/btq033.

R Core Team. *R: A Language and Environment for Statistical Computing*. R Foundation for Statistical Computing, Vienna, Austria, 2024. URL `https://www.R-project.org/`.

The ENCODE Project Consortium. An integrated encyclopedia of DNA elements in the human genome. *Nature*, 489(7414):57–74, Sept. 2012. ISSN 1476-4687. doi: 10.1038/nature11247.

H. Wickham. *ggplot2: Elegant Graphics for Data Analysis*. Springer-Verlag New York, 2016. ISBN 978-3-319-24277-4.

Y. Zhang, T. Liu, C. A. Meyer, J. Eeckhoute, D. S. Johnson, B. E. Bernstein, C. Nusbaum, R. M. Myers, M. Brown, W. Li,

and X. S. Liu. Model-based Analysis of ChIP-Seq (MACS). *Genome Biology*, 9(9), Sept. 2008. ISSN 1474-760X. doi: 10.1186/gb-2008-9-9-r137.
